# Supplementary material for: Modeling glioblastoma heterogeneity as a dynamic network of cell states
Source: Mol Syst Biol. 2021 Sep 16;17(9):e10105. doi: 10.15252/msb.202010105 (PMC8444284; doi:10.15252/msb.202010105)
Supplement: Supplementary file 6 — Source Data for Figure 5 [file MSB-17-e10105-s004.zip › Figure5A_sourcedata/GSEA_3071/nosigdb_stateV.GseaPreranked.1622795159954/SUVA_G1S_PMID_31327527.html]

Details for gene set SUVA\_G1S\_PMID\_31327527[GSEA]

|  || Dataset | state43071 |
| Phenotype | NoPhenotypeAvailable |
| Upregulated in class | na\_pos |
| GeneSet | SUVA\_G1S\_PMID\_31327527 |
| Enrichment Score (ES) | 0.8408332 |
| Normalized Enrichment Score (NES) | 3.3098915 |
| Nominal p-value | 0.0 |
| FDR q-value | 0.0 |
| FWER p-Value | 0.0 |
Table: GSEA Results Summary

  

Fig 1: Enrichment plot: SUVA\_G1S\_PMID\_31327527      
 Profile of the Running ES Score & Positions of GeneSet Members on the Rank Ordered List

  

| PROBE | GENE SYMBOL | GENE\_TITLE | RANK IN GENE LIST | RANK METRIC SCORE | RUNNING ES | CORE ENRICHMENT || 1 | HIST1H4C |  |  | 1 | 2.442 | 0.0700 | Yes |
| 2 | PCNA |  |  | 10 | 1.908 | 0.1233 | Yes |
| 3 | MCM4 |  |  | 17 | 1.815 | 0.1743 | Yes |
| 4 | RAD51AP1 |  |  | 22 | 1.757 | 0.2241 | Yes |
| 5 | CCNE2 |  |  | 32 | 1.664 | 0.2702 | Yes |
| 6 | RRM2 |  |  | 34 | 1.660 | 0.3178 | Yes |
| 7 | MCM3 |  |  | 49 | 1.586 | 0.3607 | Yes |
| 8 | GINS2 |  |  | 57 | 1.528 | 0.4033 | Yes |
| 9 | MCM7 |  |  | 88 | 1.400 | 0.4378 | Yes |
| 10 | RRM1 |  |  | 99 | 1.364 | 0.4751 | Yes |
| 11 | CENPM |  |  | 114 | 1.337 | 0.5109 | Yes |
| 12 | CDCA5 |  |  | 123 | 1.326 | 0.5475 | Yes |
| 13 | FEN1 |  |  | 139 | 1.296 | 0.5819 | Yes |
| 14 | GMNN |  |  | 146 | 1.280 | 0.6175 | Yes |
| 15 | CENPK |  |  | 162 | 1.241 | 0.6504 | Yes |
| 16 | ZWINT |  |  | 204 | 1.166 | 0.6761 | Yes |
| 17 | CDC45 |  |  | 228 | 1.138 | 0.7045 | Yes |
| 18 | ASF1B |  |  | 237 | 1.130 | 0.7354 | Yes |
| 19 | MCM2 |  |  | 238 | 1.130 | 0.7679 | Yes |
| 20 | AURKB |  |  | 297 | 1.068 | 0.7876 | Yes |
| 21 | DUT |  |  | 314 | 1.050 | 0.8147 | Yes |
| 22 | TMEM106C |  |  | 334 | 1.034 | 0.8408 | Yes |
| 23 | PKMYT1 |  |  | 506 | 0.859 | 0.8331 | No |
| 24 | TK1 |  |  | 591 | 0.806 | 0.8403 | No |
| 25 | RNASEH2A |  |  | 859 | 0.674 | 0.8090 | No |
| 26 | MELK |  |  | 915 | 0.653 | 0.8173 | No |
| 27 | CKS1B |  |  | 1502 | 0.479 | 0.7199 | No |
Table: GSEA details [plain text format]

  

Fig 2: SUVA\_G1S\_PMID\_31327527: Random ES distribution      
 Gene set null distribution of ES for **SUVA\_G1S\_PMID\_31327527**
